# One-year change in handgrip strength in patients with hip fracture: a prospective comparison with hip disease

**DOI:** 10.1186/s12877-023-03782-9

**Published:** 2023-02-02

**Authors:** Chul-Ho Kim, Jeongae Han, Ji Wan Kim

**Affiliations:** 1 Department of Orthopedic Surgery, Asan Medical Center, University of Ulsan College of Medicine, Seoul, Republic of Korea; 2 Department of Orthopedic Surgery, Chung-Ang University Hospital, Chung-Ang University College of Medicine, Seoul, Republic of Korea; 3 University of Ulsan College of Medicine, Seoul, Republic of Korea

**Keywords:** Sarcopenia, Hip fracture, Muscle

## Abstract

**Background:**

Handgrip strength (HGS) has been adopted as one of the diagnostic tools for sarcopenia and is gaining attention because of its association with osteoporotic hip fractures. Longitudinal data of HGS at multiple follow-up intervals in older hip fractures are lacking. We aimed to investigate and compare the HGS changes in patients with hip fracture within 1-year with those in patients with hip diseases.

**Methods:**

This prospective study was conducted between June 2018 and July 2020. The HGS was measured preoperatively, at predischarge, and at 3, 6, and 12 months postoperatively. We prospectively compared the number of patients with low muscle strength (LMS) as well as the HGS changes over time between the two groups.

**Results:**

A total of 115 consecutive patients with hip fracture (*n* = 58) and hip disease (57) were enrolled. The rate of preoperative LMS was higher in the hip fracture group than control (*P* = 0.005), but there was no significant difference in the postoperative period (*P* = 0.343). The mean HGS was lower in the hip fracture group at all measured time periods. The preoperative HGS increased right before discharge (15.2 kg to 17.0 kg), and plateaued thereafter 1-year in the fracture group, whereas there were no statistically significant changes in serial follow-up trends in the control group.

**Conclusions:**

The preoperative HGS in fracture patients may have been underestimated, due to different position of the arm, insufficient practice, or pain. Subsequently, HGS was rather constant during 1-year indicating no development of general sarcopenia after treatment for hip fracture. Therefore, in hip fracture patients, the predischarge HGS might be more reliable than preoperative HGS.

## Introduction

In recent times, hip fractures in older adults have posed a significant global health burden [1]. With the elderly population continuously rising not only in the US, but also in Asia, hip fractures are also increasing: a trend which is in-line with the predicted 1.4-fold increase from 2016 to 2025 [2]. Handgrip strength (HGS) has been adopted as one of the diagnostic tools for sarcopenia and is gaining attention because of its relationship with osteoporotic hip fractures. Additionally, it has been highlighted as an easy and efficient modality for predicting the prognosis and degree of functional recovery after hip fractures [3–5].

HGS measurement protocols were used as predictors of hip fractures. HGS was assessed at various time points [6], but no consensus or protocol has been established. Although some researchers measured the HGS of patients with hip fracture immediately after hospital admission based on the maintained muscle mass during the acute post-fracture stage [6–9], other studies predicted the hip fracture risk or investigated the prognosis of hip fracture by using HGS data after surgery [10, 11]—which showed controversial results. Recently, Han et al. reported that pre- and postoperative HGS reflected functional outcomes following hip fracture. They favored the postoperative HGS compared to preoperative HGS due to the higher prognostic value [5]. Although some authors have reported a relationship between lower HGS and hip fracture risk or lower functional recovery after surgery [6, 8, 9], there have also been contradicting results refuting the value of HGS in predicting hip fracture outcomes [12].

Despite this, there are no previous studies prospectively evaluating HGS longitudinally at multiple follow-up intervals, including both preoperative and postoperative periods. Therefore, this study aimed to investigate serial HGS changes within 1 year of follow-up in patients with osteoporotic hip fracture and compared these changes with those of patients with hip diseases.

## Methods

### Study design and patient recruitment

A prospective longitudinal observational study was conducted. Informed consent was obtained from all subjects involved in the study. The inclusion criteria for hip fracture patients were as follows: 1) presence of hip fractures, 2) low-energy injury, and 3) age ≥ 60 years, with prior surgery at Asan Medical Center by a single surgeon between June 2018 and July 2020. The institutional review board of the center approved the study protocol (2018–0932). Considering other potential factors that could affect any unexpected lower HGS measurements and to avoid any potential selection bias, the exclusion criteria were set as follows: 1) concomitant upper extremity injuries; 2) diagnosed malignant diseases, which might affect bias in evaluating sarcopenia due to general weakness; 3) severe cognitive disorders that make it difficult to obtain patient-reported outcome (PRO) data (Severe cognitive disorder, dementia, or severe cognitive impairment were detected by the medical history of the patient, and the conversation status level of patients was judged with the history taking; we did not exclude the patients with prior diagnosis of mild cognitive disorder); and 4) loss to follow-up or missed postoperative HGS measurements within 1-year, or patient’s death. The control group included patients who had undergone hip surgeries due to hip diseases, such as primary/secondary hip osteoarthritis, femoral head osteonecrosis, and prosthetic joint infection.

### Intervention procedures (treatment details)

For the hip fracture group, arthroplasty or osteosynthesis using cephalomedullary nails was performed. In the control group, patients diagnosed with osteonecrosis of the femoral head or hip osteoarthritis underwent arthroplasty. The patients who diagnosed implant loosening following index arthroplasty surgeries or prosthetic joint infection underwent revision surgeries or incision & drainage surgeries. There were the patients who underwent implant removal surgeries due to the implant irritation after osteosynthesis. The patients who diagnosed refractory trochanteric bursitis underwent bursectomy. All patients followed the same standardized postoperative rehabilitation program and were encouraged to practice early assisted ambulation. All patients underwent ankle pump as often as every 5 or 10 min during the admission period, and abduction exercise was performed by sliding the leg out to the side and back 10 times in 90 s 3 to 4 sessions per day. Quadriceps sets were also supervised 10 times during the 10-min period until fatigue was felt by the participant in thigh muscle. Patients underwent wheelchair ambulation on the first postoperative day, whereas standing exercises and full weight-bearing exercises with a walking aid (walker or crutches) were supervised by a physiatrist and a therapist. In our protocol, assisted full weight-bearing was defined as the weight-bearing level tolerated by the patient on their affected leg, which was usually between 50 and 100% body weight. The patients were generally discharged 5–6 days after surgery and were followed up with after 4 weeks, and at 3, 6, and 12 months.

### Variables

We investigated the patients’ age, sex, height, weight, body mass index (BMI), diagnoses, time interval from admission to surgery, and hospitalization periods. We also compared preoperative and postoperative pain using the visual analog scale (VAS). The low muscle strength (LMS) rates determined from HGS measurement, the Asian Working Group for Sarcopenia (AWGS) defined sarcopenia as age-related loss of muscle mass which could be measured “LMS”, therefore we compared the preoperative and predischarge LMS rates of the two groups by applying the recommended diagnostic cutoff value of LMS from the 2019 AWGS [13], and considering patient weights of < 28 kg in men and < 18 kg in women as major associated factors of sarcopenia.

### Measurement of HGS

HGS was measured following the 2019 AWGS recommendation [13] using a Takei digital grip strength dynamometer (Model T.K.K.5401, Takei, Niigata, Japan). We measured the HGS on both hands and used the best performance among the three trials in a maximum-effort isometric contraction. Basically, HGS was measured in the sitting position with 90° elbow flexion, whereas in the hip fracture group with limited sitting position due to pain during the preoperative period, the HGS was measured in a supine position. After the hip fracture surgery, all HGS data were obtained in a sitting position. The HGS was measured at initial ward admission (preoperative), at 3–4 days after surgery (before discharge, predischarge), and 3, 6, and 12 months postoperatively. To avoid the bias from the HGS measurement, all data collection was performed by a well-trained clinical research nurse throughout the admission periods for each patient.

### Statistical analysis

The independent t-test and chi-square test were used for continuous and categorical variables, respectively. The HGS trends of the two groups were compared using repeated analysis of variance tests. The independent t-test was used to compare the findings of both groups at each follow-up. We performed the repeated analysis of variance between the hip fracture and control groups. Post-hoc analysis using the Bonferroni method was also used to compare the results between the time points within each group to adjust the result of repeated analysis of variance. The power for the primary endpoint was calculated by considering preoperative HGS differences between the two groups. Using the mean HGS and standard deviation, a power of 0.9, and a 5% statistical significance (alpha), a population of at least 30 participants per group was required. All statistical analyses and interpretations were performed by the professor of the department of applied statistics in the university and the orthopedic faculty, who have prior experience of performing several statistical analyses (C.-H.K.).

## Results

### Demographic variables

**Fig. 1:**
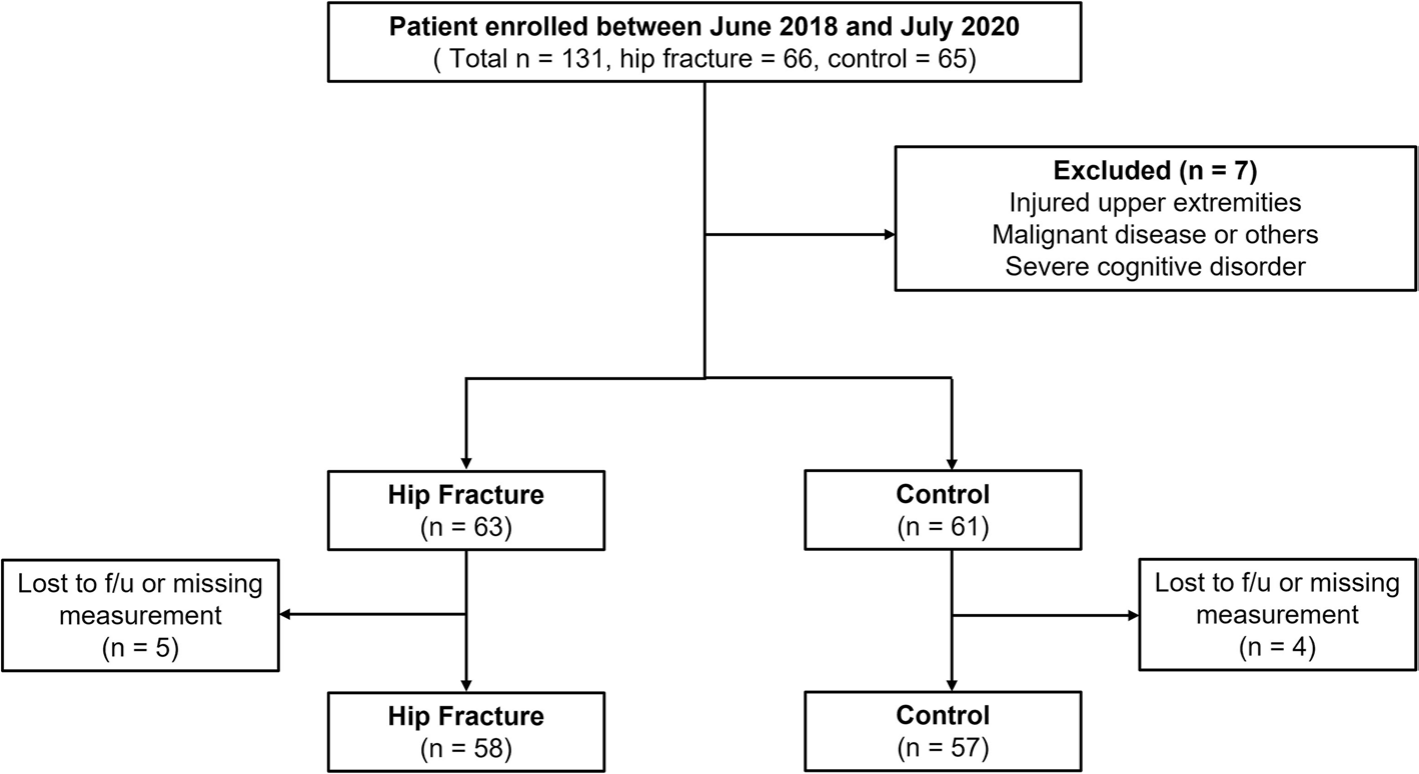
Flow diagram of the patients included in the study

After excluding seven patients (two patients with injured upper extremities, two malignant disease patients with general weakness, and three patients with cognitive disorder; because of difficulty in collecting reliable clinical data), 63 patients were screened in the hip fracture group and 61 in the control (hip disease) group. Patients who were lost to follow-up, including those who died within 1-year, were also excluded. Finally, a total of 115 consecutive patients (58 with hip fractures and 57 with hip diseases) were included in this study (Fig. 1). All consecutive patients meeting the inclusion criteria were enrolled. The hip fracture group was older on average (76.1 ± 7.4 years; range, 61–90 years) than the control group (68.6 ± 6.6 years; range, 60–85 years; *P* < 0.001), although there was a similar representative population of men and women (*P* = 0.055). BMI was lower in the hip fracture group (*p* = 0.009). The mean time from admission to surgery was 2.6 days and 2.2 days in the hip fracture and control groups, respectively, with no significant difference (*P* = 0.143). The average hospital stay was 10.2 days and 8.7 days in the hip fracture and control groups (*P* = 0.160), respectively. Femoral neck fracture was the most common hip fracture, whereas femoral head osteonecrosis was the most common non-fracture diagnosis. Further details are presented in Table 1. The hip fracture group showed higher preoperative and lower postoperative VAS scores than the control group (*P* = 0.049 and *P* < 0.001, respectively).

**Table 1:** Patient demographics between hip fracture and control group

Variables	Data		*P*-value
	Hip fracture (*n* = 58)	Control (*n* = 57)	
Age, years ± SD (range)	76.1 ± 7.4 (61–90)	68.6 ± 6.6 (60–85)	** < 0.001**
Female sex, n (%)	48 (82.8%)	38 (66.7%)	0.055
BMI, kg/m^2^ ± SD (range)	23.6 ± 3.8 (15.2–35.7)	25.4 ± 3.6 (19.6–34.7)	**0.009**
Admission–surgery, days ± SD (range)	2.6 ± 2.3 (1–17)	2.2 ± 0.5 (2–5)	0.143
Hospital stay, days ± SD (range)	10.2 ± 7.3 (5–56)	8.7 ± 3.5 (6–25)	0.160
Preoperative VAS score ± SD (range)	3.5 ± 1.8 (1–7)	3.0 ± 0.7 (2–6)	**0.049**
Predischarge VAS score ± SD (range)	1.6 ± 0.9 (0–3)	2.3 ± 0.8 (0–5)	** < 0.001**
Preoperative LMS patients, n (%)	47 (81.0%)	32 (56.1%)	**0.005**
Predischarge LMS patients, n (%)	38 (65.5%)	32 (56.1%)	0.343
Preoperative diagnosis	Femur neck Fx.: 29 (50%) Intertrochanteric Fx.: 21 (36.2%) Subrochanteric Fx.: 8 (13.8%)	ONFH: 19 (33.3%) 2’ OA: 13 (22.8%) 1’ OA: 9 (15.8%) PJI: 3 (5.3%) Etc.: 13 (22.8%)	
Surgical treatment	Primary arthroplasty: 29 (50%) Osteosynthesis: 29 (50%)	Primary arthroplasty: 45 (78.9%) Revision arthroplasty: 3 (5.3%) Implant removal: 4 (7.0%) I&D: 4 (7.0%) Bursectomy: 1 (1.8%)	

*BMI* Body mass index, *Fx.* Fracture, *LMS* Low muscle strength, *OA* Osteoarthritis, *ONFH* Osteonecrosis of femoral head, *PJI* Prosthetic joint infection, *VAS* Visual analog scale, *SD* Standard deviation

*P*-values < 0.05, marked in bold indicate statistical significance

### The serial trends and comparisons of HGS between hip fracture group and control group

The preoperative and predischarge LMS rates in the hip fracture group were 81.0% and 65.5%, respectively. In the control group, both preoperative and predischarge LMS rates were 56.1%. Although the preoperative LMS rate of the hip fracture group was significantly higher (*P* = 0.005), the predischarge rates between the two groups were not significantly different (*P* = 0.343).

**Fig. 2:**
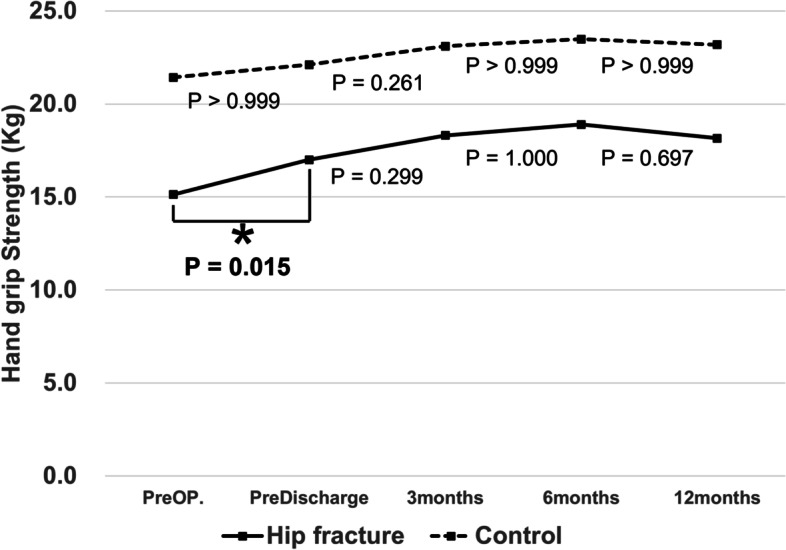
Multiple follow-up trends of handgrip strength on both the hip fracture and control groups. The preoperative baseline handgrip strength increased at predischarge (asterisk)

The serial HGS follow-up trends in both groups are shown in Fig. 2. The mean HGS was lower in the hip fracture group than in the control group at all time points. The preoperative baseline HGS increased at predischarge from 15.2 kg to 17.0 kg (*P* = 0.015), and plateaued within 1 year after surgery. However, there were no statistically significant changes in serial HGS follow-up trends in the control group. Additional details are presented in Table 2.

**Table 2:** Changes of hHandgrip strength in two groups over time

Variables	Handgrip strength		*P*-value*
	Hip Fracture Group	Control Group	
Preoperative	15.2 ± 7.2 kg (2.6–39.9)	20.7 ± 7.8 kg (9.0–40.5)	** < 0.001**
Predischarge	17.0 ± 8.0 kg (2.6–42.4), ^†^***P* = 0.015**	21.2 ± 8.6 kg (9.5–43.4), ^†^*P* > 0.999	**0.002**
Month 3	18.3 ± 8.1 kg (7.0–48.7), ^†^*P* = 0.299	22.8 ± 8.0 kg (9.7–42.6), ^†^*P* = 0.069	**0.001**
Month 6	18.9 ± 7.4 kg (8.0–42.8), ^†^*P* = 1.000	23.1 ± 8.1 kg (10.8–44.3), ^†^*P* > 0.999	**0.002**
Month 12	18.1 ± 7.5 kg (5.4–42.1), ^†^*P* = 0.697	22.9 ± 7.7 kg (12.0–43.8), ^†^*P* > 0.999	** < 0.001**

Original *P*-value = 0.373 (*P*-value, difference between groups over time) (repeated analysis of variance)

^†^Comparison with previous follow-up point

^*^Comparison between the two groups at each follow-up point (independent t-test)

*P*-values < 0.05, marked in bold indicate statistical significance

## Discussion

The current study compared serial HGS changes between the hip fracture and control groups from the preoperative to the postoperative period. In the hip fracture group, the preoperative HGS increased at predischarge, but plateaued by the 3-, 6-, and 12-month measurements. In contrast, there were no statistically significant changes in serial HGS follow-up trends in the control group. The HGS did not decrease after surgery in either group.

Our results showed the importance of optimal timing when administering the HGS test in patients with hip fractures. To date, there is no evidence regarding the optimal HGS measurement time in patients with hip fractures, although there has been a consensus that HGS measurement could predict the prognosis of hip fractures. Selakovic et al. [4] highlighted the importance of HGS measurement in the acute post-hip fracture stage, and Savino et al. [9] measured the HGS at hospital admission to predict walking recovery within 12 months after hip fracture onset. Alvarez et al. [8] recommended HGS assessment within the first hour of hospital admission for hip fracture surgery, as an indicator of functional recovery after 3 months. D’ Adamo et al. and Fox et al. reported maintained muscle mass in the first 10 days after hip fracture, before it subsequently diminishes [14, 15]; therefore, baseline HGS should be measured as early as possible after hip fracture [6, 8, 9], even though it is difficult to maintain a standardized protocol for measuring HGS in the sitting position. Interestingly, our serial results revealed that the preoperative HGS in patients with hip fracture was lower than that in patients with predischarge or postoperative HGS. The time interval between preoperative and predischarge HGS measurements was < 10 days in the hip fracture group. Muscle mass has been reported to be maintained during the first 10 days after hip fracture [14, 15], before significantly decreasing afterwards. Comparing these results with ours, theoretically, it does not make sense that muscle strength increases—especially during admission after hip fracture. In contrast, there were no differences between the preoperative and pre-discharge HGS in the control group.

The lower preoperative HGS in hip fracture patients could be due to the following reasons: 1) hip fracture pain may cause underestimated preoperative HGS, which is supported by the fracture group showing higher preoperative VAS scores that improved more than the VAS scores of the control group after surgery; 2) differences in the HGS-measuring methods could cause bias or underestimated preoperative HGS. Moreover, there were controversies about the testing position and the result of HGS measurement. Teraoka [16] reported that HGS in the supine position was weaker than that in the standing or sitting position, suggesting the influence of gravity. Hillman et al. [17] also reported that the HGS was significantly stronger in a sitting position than in a supine or armchair position. Considering the difficulty and inevitability in adhering to the standard HGS measurement protocol in the acute post-fracture stage, the authors recommend a predischarge HGS as an optimal baseline parameter to reflect muscle strength in patients with hip fracture. Indeed, there were a number of protocols that were most frequently used, such as ASHT or the Southampton protocol, which also recommend measuring HGS in a sitting position [3, 18]. We believe that our current results could be helpful in establishing a HGS measuring protocol to predict the prognosis of hip fractures in older adults.

Our results showed that HGS was maintained within 1-year after surgery, which was different than expected. Clinically, we often encounter patients with lower limb weakness after hip fractures. Pham et al. [19] reported on muscle weakness after hip fracture, which showed as an 18% increase in post-fracture mortality risk in post-fracture quadriceps strength weakness in a 25-year prospective study of 1,184 patients. In contrast, we could not identify HGS weakness during the follow-up in either group. This is comparable to the results of Visser et al. [20], who reported decreased ankle dorsiflexion strength and no significant change in grip strength between baseline and 12 months after hip fracture surgery in Caucasian female patients aged ≥ 65 years. In our opinion, the use of a walker or crutches for ambulation recovery exercises after hip fracture could encourage the use of arm or handgrip muscles, maintaining HGS.

Despite no statistical difference in the overall HGS trends in both groups (*P* = 0.373), the mean HGS was lower at all measured time periods in the hip fracture group. Comparing the demographic variables, the mean age was older and the BMI was lower in the hip fracture group. Considering this, our results make sense. A relatively low HGS, which is also related to sarcopenia, has been observed in patients with hip fractures. Coupland et al. [21] reported in their population-based age- and sex-matched study that the decline in HGS significantly increased the risk of hip fracture. Cawthon et al. [22] also reported that older men who were unable to complete the HGS measurement showed a higher risk of hip fracture, suggesting LMS. Recently, Denk et al. [23] also reported a negative relationship between HGS and hip fracture risk in their systematic review of 11 studies, which is comparable to the findings of our study.

Previously, there were some efforts to reveal the minimum clinically important difference (MCID) for HGS. However, in the current study, we did not adopt the MCID to interpret our study results since the studies suggesting this value vary. According to the recent review article by Bohannon [24], the MCID of HGS is studied as ranging from 0.04 to 6.9 kg. In our study, the difference in HGS ranged from 0.2 to 1.8 kg following each measuring time point. Another study, [25] conducted in the same country as ours, that evaluated MCID, revealed MCID as 5.0 to 6.5 kg; however, this study was performed for the distal radius fracture patients and not for the hip fracture, and the mean age of patients was 55 years (range, 26–68 years).

The optimal HGS measurement time in patients with hip fractures could also be related to the rehabilitation performed following surgery. Recently, some studies have focused on this issue. A retrospective study by Neuerberg et al. [26] reported the superior recovery and treatment outcomes with appropriate rehabilitation in an integrated orthogeriatric care center and compared them to those conducted in a conventional trauma care center. Additionally, other recent papers have shown similar results [27, 28], highlighting the importance of proper postoperative rehabilitation to achieve satisfactory recovery status for elderly hip fracture patients. Therefore, proper postoperative management focusing on patient-tailored rehabilitation after hip fracture should be performed, since this could affect not only the HGS measurement data but also the optimal HGS measurement timing.

This study has several limitations. First, despite a sufficient number of patients for our prospective study, we could not perform a matched controlled study because of the difficultly in matching groups according to age and sex. Indeed, the study and control groups varied by age and sex (Table 1). Therefore, a selection bias would emerge when comparing the two groups. However, to the best knowledge of our knowledge, this is the first study to prospectively investigate the serial follow-up trends of HGS in hip fractures among the elderly, and it also included more study materials compared to those in previous studies that reported MCID in HGS of patients with wrist fracture. Second, we measured HGS in a sitting position at all time periods in both the hip fracture and control groups, although preoperative HGS in hip fracture patients was inevitably measured in a supine position due to the pain. This could cause a potential bias when interpreting the study results. Third, we could not perform functional evaluation and outcome prediction matched with the HGS trends. However, our prospective study is the first to show multiple follow-up results of the comparison of HGS between patients with hip fracture and those with hip disease. Our results may help to establish further studies on HGS in patients with hip fractures.

## Conclusions

The preoperative HGS in fracture patients may have been underestimated, due to different position of the arm, insufficient practice, or pain. Subsequently, HGS was rather constant during 1-year indicating no development of general sarcopenia after treatment for hip fracture. Therefore, in hip fracture patients, the predischarge HGS might be more reliable than preoperative HGS.

## Data Availability

The data presented in this study are available on request from the corresponding author. The data are not publicly available due to conditions of the ethics committee of our university.

## Abbreviations

HGS: Handgrip strength
PRO: Patient-reported outcome
BMI: Body mass index
VAS: Visual analog scale
LMS: Low muscle strength
AWGS: The Asian Working Group for Sarcopenia
